# Using Citizen Science and Field Surveys to Document the Introduction, Establishment, and Rapid Spread of the Bare-Eyed Pigeon, *Patagioenas corensis*, on the Island of Saint-Martin, West Indies

**DOI:** 10.3390/biology13080585

**Published:** 2024-08-01

**Authors:** Christopher Cambrone, Anthony Levesque, Frank Cézilly

**Affiliations:** 1 Caribaea Initiative, Le Raizet, 97139 Les Abymes, Guadeloupe, France; frank.cezilly@caribaea.org; 2 Association Amazona, Pointe d’Or, 97139 Les Abymes, Guadeloupe, France; anthony.levesque@wanadoo.fr

**Keywords:** biological invasion, Caribbean, Columbidae, eBird, wildlife trade

## Abstract

**Simple Summary:**

Early detection of the introduction and establishment of exotic species is crucial to prevent further spread and minimize negative impacts on native species, ecosystems, and agrosystems. Using citizen science and field surveys, this study documents the establishment and expansion of the Bare-eyed Pigeon, *Patagioenas corensis*, native to South America, on the island of Saint-Martin, the West Indies. Following its introduction about 10 years ago, the species is now expanding fast on the island, where it could compete with native bird species. First observed in coastal areas, the Bare-eyed Pigeon has progressively diversified its habitat use to exploit anthropized areas. Evidence for steady increases in both numbers and reproductive activity on the island calls for rapid management before it is too late. To that end, we strongly recommend adding the exotic Bare-eyed Pigeon to the official list of game species on Saint-Martin.

**Abstract:**

Early reporting of the introduction and establishment of exotic species is of paramount importance for successful management. Here, we report the introduction and rapid spread of the Bare-eyed Pigeon, *Patagioenas corensis*, on the binational island of Saint-Martin, the West Indies. This range-restricted species naturally occurs in arid coastal areas of Columbia and Venezuela and nearby islands. Its introduction on Saint-Martin represents an expansion of about 1000 km beyond its established native range. Using observations recorded in eBird and results from a recent field survey, we show that since its introduction, most probably between late 2012 and early 2013, the species has expanded fast in Saint-Martin and has recently broadened its habitat to include anthropized, built areas. The expansion of Bare-eyed Pigeon on Saint-Martin and the neighboring Leeward Islands, possibly facilitated by climate change in the future, could be a threat to both native columbid species and other bird species through competition for resources. We therefore recommend that local authorities and stakeholders rapidly eradicate the species or at least prevent its further spread on Saint-Martin, possibly though listing it as a game species, while it is still possible to do so.

## 1. Introduction

The introduction and spread of exotic species can severely affect ecosystems, agriculture, health, and biodiversity, particularly in insular environments [1,2,3], and might be accelerated in the coming decades by change in both land use and climate [4,5]. Because of the huge ecological and economic costs associated with biological invasions [6,7], especially in insular ecosystems [8], preventing the introduction or establishment of exotic species is a major concern today for conservation biologists [9].

Although exotic species can be introduced through multiple pathways, trade and transport are regarded as the major drivers of introduction on islands, while land use/cover change appears to be the main determinant of their establishment and spread [9]. Consequently, several countries and small developing island states have reinforced their control at points of entry [10], but only with limited success as records of new exotic species on islands are still regularly reported [11,12,13,14]. Because, in most cases, the eradication of exotic species on islands is possible only soon after the onset of the invasion process, early reporting of the introduction and establishment of exotic species on islands is of paramount importance for the successful management of biological invasions [15]. The early detection of invasive species can be enhanced through various techniques, such as environmental DNA, chemoreception, or remote sensing [16]. More recently, the combination of citizen science with professional expertise has been identified as an efficient way of documenting the introduction, establishment, and expansion of exotic species [16,17]. This is particularly true of invasive or expanding bird species that can be easily observed and identified by amateur ornithologists, as illustrated by several studies [18,19,20].

Here, we document the recent introduction, establishment, and rapid expansion of the Bare-eyed Pigeon, *Patagioenas corensis*, originating from north-west South America, on the binational island of Saint-Martin, the West Indies, about 1000 km beyond its native range. Using a combination of citizen science and field survey, we quantified the demographic and spatial expansion of the species on the island based on the chronology of reported observations in eBird and characterized its habitat occupancy. We discuss the potential consequences of this invasion at the local and regional levels and make recommendations for rapid management actions.

## 2. Materials and Methods

### 2.1. Study Area

The island of Saint-Martin (hereafter called Saint-Martin) lies between 18.0° and 18.1° north latitude and between 63.2° and 63.0° west longitude, approximately 300 km east of Puerto Rico. It belongs to the Leeward Islands, a group of islands with similar ecological characteristics situated where the northeastern Caribbean Sea meets the western Atlantic Ocean. The island covers about 87 km^2^ and is rather flat, with a mean elevation of about 34m and maximum elevation of 424 m a.s.l. (Pic Paradise, in the center of the island). It is administratively divided into two parts. The northern part (55 km^2^), called Saint-Martin, is a French overseas territory, while the southern part (34 km^2^), called Sint Maarten, is a Dutch overseas constituent country. The local climate is characterized by stable temperatures (25–28 °C throughout the year) and seasonally variable precipitation [21]. The island landscape consists of undulating coastline with sandy beaches and impounded salt ponds, offshore islets, and dry landscapes, with some rain-dependent vegetation at higher elevation [21]. According to WWF [22], Saint-Martin comprises two major ecoregions: Leward islands xeric shrubs and Lesser Antilles mangroves.

### 2.2. Study Species

The Bare-eyed Pigeon has overall pale, sandy-colored plumage; a large pale blue ocular ring with a wider outer area of reddish brown; orange to orange–brown iris; and conspicuous white crescent wing patches contrasting with black flight feathers [23]. The head, neck, and breast are mauve–pink, with blue–grey on top of the head and a pale pinkish bill. Neck feathers are edged with some pinkish-bronze, black bands, and pale brown margins, producing a scaled effect. Back, rump, and tail are grey, while feet are pink. Its distinctive appearance makes the species easy to identify in the field. However, although much larger, it can be confused with the White-winged Dove, *Zenaida asiatica*, especially young individuals. However, it has a distinctive call, consisting of two short chucks interspersed between two loud and melodious notes [24].

The species is most often seen in pairs or trios but can form small flocks of about 10-20 individuals and even be part of mixed-species flocks with other columbid species [24]. It mainly occurs in arid lowlands with shrub-like vegetation but can also be observed in mangroves and cultivated areas [25]. However, it is increasingly present in residential areas and around the grounds of hotels and resorts on Aruba, Bonaire, and Curaçao [26]. It feeds upon various seeds and fruits, including cultivated plants [23,25]. Breeding pairs build rudimentary nest platforms made of twigs in trees, bushes, and cacti, where the female lays a single-egg clutch. In its native range, the species is essentially sedentary, with occasional flock movements in relation to food availability.

### 2.3. Data Collection

We compiled all records of the Bare-eyed Pigeon on Saint-Martin to track its introduction history and spread on the island, relying on all observations of the species on the island available on eBird, from 2013 (corresponding to the first reported observation) up to 2024 [27]. We checked for double-count events, corresponding with two different observers independently reporting the same observation (i.e., same place, same date, and same time). As a control, we take the same action for the Zenaida Dove, *Zenaida aurita*, a resident species and one of the most abundant native Columbidae on Saint-Martin. Records from eBird included our own observations of the two species made during a recent two-day field survey conducted from 5 to 7 July 2023, during which we covered 175 km, mainly on the French part of the island (see Supplementary Materials: Table S1) and proceeded to 40 separate 5-min auditory and visual counts to record the presence and abundance of the two species. We also made casual observations of Bare-eyed Pigeons on some occasions during our presence on the island.

### 2.4. Data Analysis

All statistical analyses were performed using the package “stats” in R software 4.4.0 [28], with a significance level set at 0.05.

To analyze variation in the spatial distribution of the Bare-eyed Pigeon through time, we considered three different periods (2015–2019, 2020–2022, and 2023–2024) to ensure at least 15 observations for each time period. We did not consider the years 2013–2014, as only one observation has been reported in eBird for that period, corresponding to the first observation of the species on Saint Marin (21 January 2013; observation list ID of eBird: S12697637). We then relied on QGIS 3.36.3 (QGIS.org [29]) to report all observations on heat maps for both the Bare-eyed Pigeon and the Zenaida Dove. The spatial density of independent observations was represented, for each species and each time period, using heat map function and circles with a radius of 1000 m around observation locations. We considered this radius size as columbid species similar in size and ecology to the Bare-eyed Pigeon tend to have a good flight ability [30,31]. We further assessed to what extent the Bare-eyed Pigeon’s distribution area on the island increased through time. To that end, we relied on a one-tailed Spearman rank correlation test to assess to what extent the distance between the location of each observation and that of the first observation of the Bare-eyed Pigeon on the island increased through time.

To test for an increase in abundance through time, we classified all observations of Bare-eyed pigeons and Zenaida doves into two groups, according to whether a single bird or more than one bird was reported. We then relied on a logistic regression (binomial generalized linear model with a logit link) to assess to what extent the probability of observing more than one Bare-eyed Pigeon or Zenaida Dove in eBird counting events was influenced by the species, the year of observation, and their interaction. We relied on quantile–quantile and goodness-of-fit plots using the R package “DHARMa” for verifying GLMM assumptions and detecting deviations from the expected distribution [32]. Because the number of observations differed markedly between the two species and the interaction was significant (see results), we relied on a Type III analysis of deviance [33,34], as implemented in the R library “car” [35] and estimated the magnitude of the effects of model parameters using odds ratios and their 95% confidence interval [36,37]. We then performed a logistic regression of the proportion of observations with more than one individual as a function of time for each species separately and tested for the significance of the models’ parameters using an analysis of deviance.

We then assessed to what extent observations of the Bare-eyed Pigeon were randomly distributed on Saint-Martin or were associated with particular habitats. To do this, we created a buffer area with a 250 m radius around all observation points. We then overlapped this buffer layer on a land cover raster file created by the European Spatial Agency (ESA WorldCover 10-m 2021 v200; [38]) to count the number of pixels (surface-like estimate) for each category of land cover around all observation points. We first considered all land cover categories characterizing Saint-Martin according to ESA WorldCover 10 m 2021 v200. However, to increase statistical power, we pooled them into four main types of habitats, including Wetland habitats (Permanent water bodies, Mangroves and Herbaceous wetlands), Open habitats (Croplands, Grasslands, Shrublands and Bare and sparse vegetation), Anthropized habitats (Built-up areas), and Tree-covered habitats (Tree cover). The number of pixels and their proportions were also estimated for each land cover category at the scale of the entire island. We then relied on a Chi-square goodness-of-fit test to assess to what extent the proportions of the different habitats around observation points differed from what would be expected under the assumption of random habitat selection, and we calculated the Cohen W effect size and its bootstrapped confidence intervals (95%; 10,000 iterations) to assess the magnitude of the difference [39]. To assess change in habitat preference over years, we reduced the multidimensionality of the land cover dataset around observation points to two major axes using principal component analysis, as implemented in the R package “FactoMineR” [40]. We then assessed to what extent the contributions of observation events to principal components were related to the year of observation, using a Spearman rank correlation test. We further calculated the diversity of habitats around observation points by relying on the Shannon index [41], using the R package “vegan” [42] to assess to what extent the diversity of habitats varied over years using a Spearman rank correlation test.

## 3. Results

**Figure 1:**
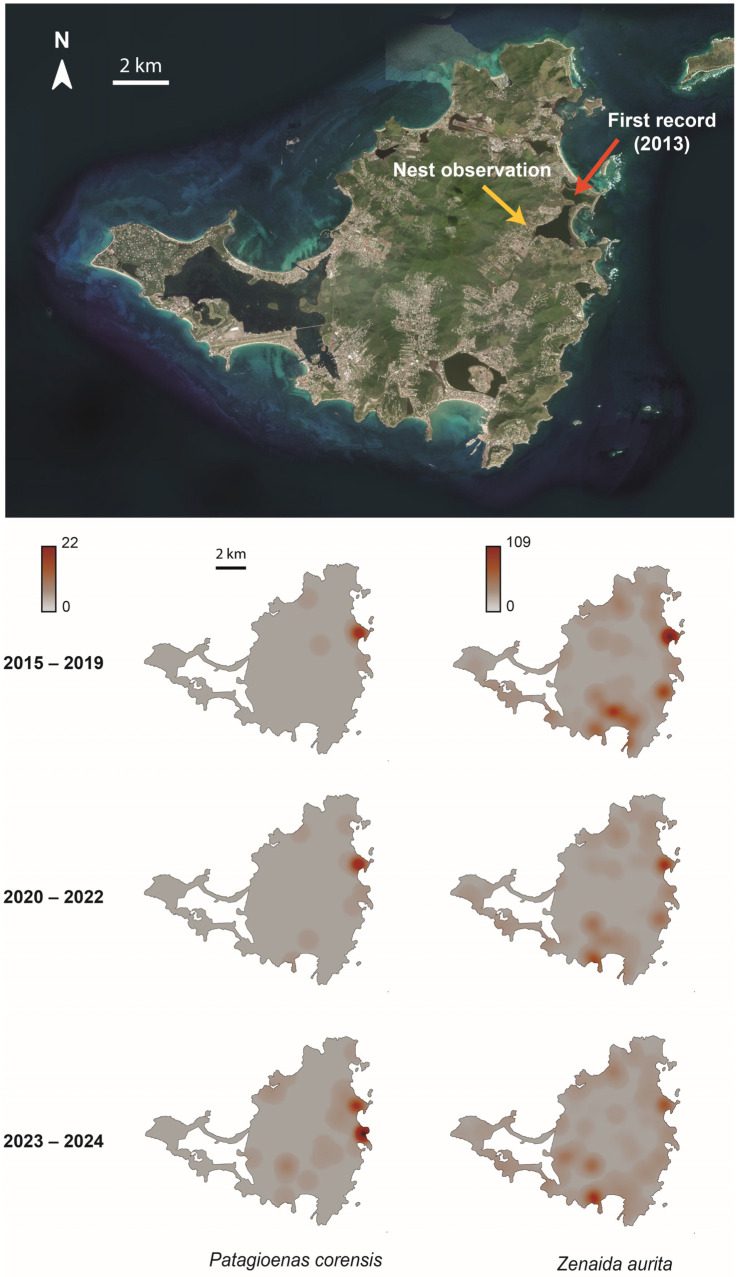
Heat maps showing spatial distribution changes for the Bare-eyed Pigeon (**left**) and the Zenaida Dove (**right**) over three time periods. The top map is a satellite view of Saint-Martin (Sources: ESRI) showing the first record of the Bare-eyed Pigeon on the 21 January 2013, indicated by a red arrow. The yellow arrow indicates the location of the nest observed on the 7 July 2023 at 03:30 p.m.

From eBird, we retrieved 108 and 2043 independent observations of the Bare-eyed Pigeon and the Zenaida Dove, respectively, for the 2013–2024 period. Figure 1 shows the spatial distribution of observations of the two species reported in eBird for the three considered time periods (i.e., 2015–2019, 2020–2022, and 2023–2024), with the invasive species most often co-occurring with the native species.

According to eBird data, the first observation of Bare-eyed Pigeon on Saint-Martin was made on 21 January 2013, in the eastern part of Saint-Martin, close to the brackish pond known as “Salines d’Orient”.

**Figure 2:**
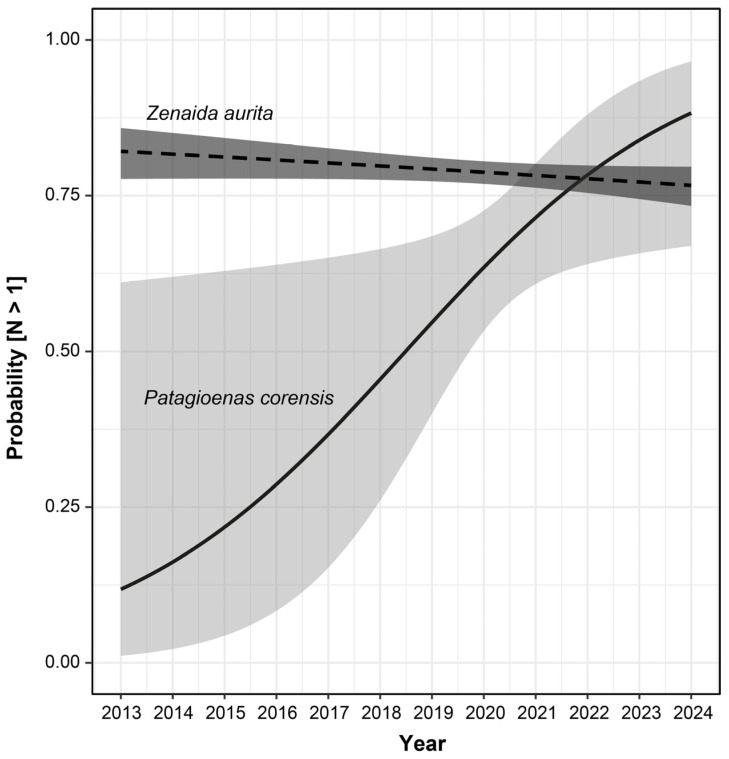
Variation in the probability of detecting more than one individual on each observation reported in eBird for *Patagioenas corensis* and *Zenaida aurita* between 2013 and 2024. The lines represent the predictive trends for each species according to model A (Table 1), with 95% confidence intervals indicated by grey ribbons around the lines (light grey: *P. corensis*, dark grey: *Z. aurita*).

Thereafter, no observation of the species was reported until 4 January 2015. Since that second observation, the number of observations continuously increased (2015–2019 = 19, 2020–2022 = 24, and 2023–2024 = 64), with an evident spatial expansion of the species over time from its supposed point of introduction across the island, first to the north and west, and later to the south (Figure 1: Spearman’s correlation test, r_s_ = 0.355, *p* < 0.0001). This spatial expansion was accompanied by an increase in the proportion of observations with more than one Bare-eyed Pigeon over time (Table 1(AC) and Figure 2). In contrast, that probability was stable or tended to slightly decrease for the Zenaida Dove (Figure 2, Table 1(B)).

**Figure 3:**
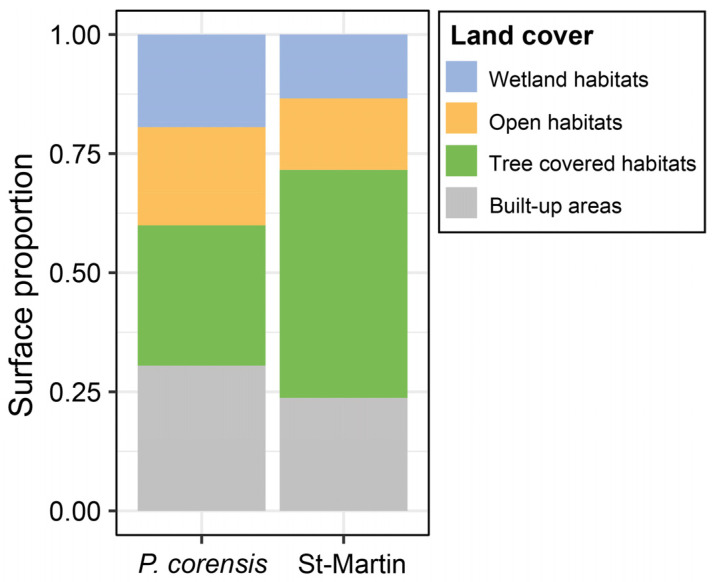
Proportion of habitat types around observation points of the Bare-eyed Pigeon reported on eBird.org and for the entire island of Saint-Martin.

**Figure 4:**
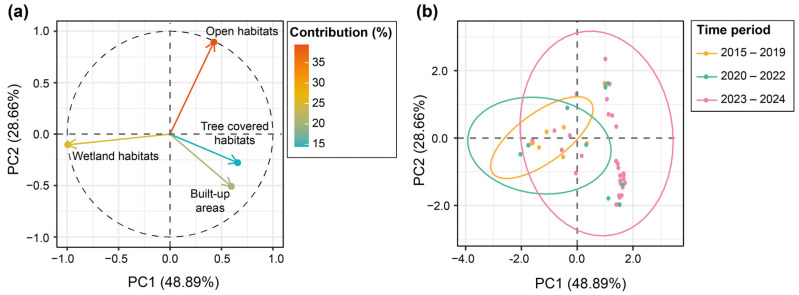
Plots showing the ACP results. Plot (**a**) displays the contributions of the four land cover types to the first two principal components (PC1 and PC2), explaining 48.89% (PC1) and 28.66% (PC2) of the total variance. See Appendix A, i.e., Table A1, for the factor loadings of the four types of land cover on the PC1 and PC2. The color gradient represents the magnitude of contribution from each land cover type, with red indicating higher contributions and green indicating lower contributions. Plot (**b**) shows the distribution of eBird observations along PC1 and PC2 for different time periods. Ellipses give a visual indication of the spread and central tendency for each time period, with a confidence level of 95%.

Although all categories of land cover characterizing Saint-Martin were found around observation points of the Bare-eyed Pigeon (Figure 3 and Figure 4), most observations were made along the coastline, corresponding mainly to wetland habitats, anthropized areas, and open habitats. Indeed, the spatial distribution of the species differed significantly from random expectation (X^2^ = 9396.1, d.f. = 3, *p* < 0.0001; Cohen’s W [95% CI] = 0.371 [0.365; 0.378]), with an over-representation of costal habitat (Figure 3).

Principal component analysis reduced the variation in land cover to two principal components, explaining, respectively, 48.89% and 28.66% of the total variation (Figure 4; see in the Appendix A, Table A1). Larger values of PC1 represented observation points in areas dominated by tree cover and anthropized habitats, while lower values corresponded to observations made in areas dominated by wetland habitat. For PC2, larger values represented observation points made in areas dominated by open habitat, while lower values corresponded to observations of pigeons in areas dominated by anthropized habitats (Figure 4). The contribution of observation points to PC1 increased significantly through time (Spearman’s correlation test, r_s_ = 0.412, *p* < 0.0001). In contrast, the contribution of observation points to PC2 was independent of time (r_s_ = −0.004, *p* = 0.970). This corresponds to the fact that Bare-eyed Pigeons were more likely to be observed in tree-covered and anthropized areas in recent times, whereas, initially, the species was mainly observed in coastal wetland habitats (Figure 4). In addition, the diversity of habitats around observation points increased significantly through time (r_s_ = 0.204; *p* = 0.018), evidencing that the Bare-eyed Pigeon diversified its habitat use during its establishment.

## 4. Discussion

Our results provide the first quantitative assessment of the invasion dynamics of the Bare-eyed Pigeon on Saint-Martin. Although the precise date of the introduction of the species on Saint-Martin cannot be firmly established, we can use observations of the Zenaida Dove to estimate a time window. Indeed, our results show that both species tend to occupy the same areas on the island (see Figure 1). In addition, the risk of the two species being confused by birdwatchers is very low, given the obvious morphological differences between them, such that we can reasonably consider that if the invasive species had been present at the same time observations of the native species were made in the past, it would very probably have been detected. We therefore estimate that the introduction of the Bare-eyed Pigeon on Saint-Martin most likely occurred between 10 October 2012 (last eBird survey made in the area of the first observation of the Bare-eyed Pigeon) or 12 December 2012 (last observation of the Zenaida Dove reported on eBird for 2012) and 21 January 2013 (the date of the first sighting of the Bare-eyed Pigeon reported on eBird).

To the best of our knowledge, there is no information on the origin of the introduction of the Bare-eyed Pigeon to Saint-Martin. The most likely hypothesis is therefore that the animals escaped accidentally or were released following voluntary introduction as pets [43]. Indeed, relatively rare pigeon species are particularly sought after by collectors (e.g., [44]) and illegal trade is considered a major threat to several of them [45]. In addition, bird species caught in the wild and offered for sale on the market have a particularly high invasion success rate [46]. The presence of the species on Aruba, Curaçao, and Bonaire [47], three other islands belonging to the Netherlands Antilles, may explain the origin of the introduced individuals. However, the captive breeding of wild pigeon species is also practiced illegally in the French West Indies (C. Cambrone pers. obs.). Specifying the origin of the introduction of the Bare-eyed Pigeon to Saint-Martin would be important to ensure that this route of arrival can be neutralized to prevent any further introduction.

**Figure 5:**
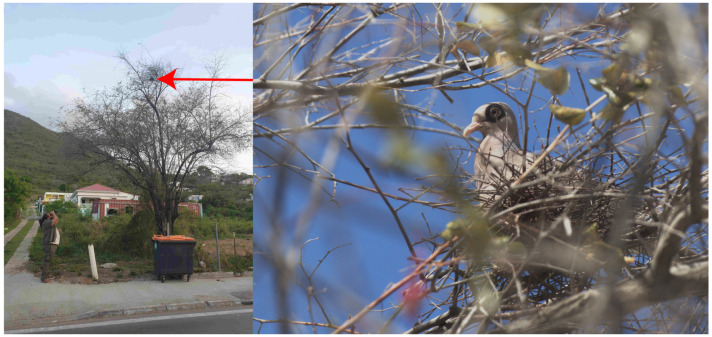
Photography of a Bare-eyed Pigeon nesting in an Indian jujube tree, *Ziziphus mauritiana*, on a very lively street (18.073410, −63.031927).

Our analysis shows that the Bare-eyed Pigeon population on Saint-Martin has increased in size and diversified its habitat use since its introduction. Interestingly, the species progressively modified its land use from traditional coastline habitats to anthropized parts of the island, including urban or suburban habitats, akin to what has been reported for other expanding Columbidae species [48,49,50,51]. For instance, the Woodpigeon, *Columba palumbus*, originally found in forested habitats, has recently expanded its habitat use to suburban parks and gardens in Algeria, similarly to what has been observed in most European countries [50]. To what extent the increase in numbers and spatial expansion of the Bare-eyed Pigeon on Saint-Martin is the consequence of the reproductive activity of a few individuals introduced on one single occasion or indicative of recurrent introductions since the first observation is unclear at the moment. However, although our field survey was of too short a duration to investigate the impacts of reproduction of the Bare-eyed Pigeon on Saint-Martin, we casually observed and photographed an individual nesting in an Indian jujube tree, *Ziziphus mauritiana*, on a busy street lined with numerous houses (Figure 5), which corresponds to the main road connecting the French and Dutch parts of the island. The location of this nest was about 300 m west of the brackish pond “Etang aux poissons” and less than two kilometers southwest of the “Salines d’Orient”, where the first observation of the Bare-eyed Pigeon was made (Figure 1). This is direct evidence of the ability of the species to reproduce, or at least attempt to, on Saint-Martin.

The establishment and rapid spread of the Bare-eyed Pigeon on Saint-Martin deserves particular attention from local and regional authorities and managers. First, although there is so far no evidence for interference competition between the Bare-eyed Pigeon and native columbid species on Saint-Martin, the invasive species may compete with them for access to food and nesting sites in the future. Second, although rare, cases of hybridization between columbid species in the wild have been reported [52], and evidence of hybridization between two *Patagioenas* species in captivity has been reported [53], such that the expansion of the Bare-eyed Pigeon may come with a risk of uncontrolled gene flow into populations of native related species. Third, the species tends to be quite common and ubiquitous in its natural distribution area, including around human settlements [47]. Given the recent intensification of urbanization on Saint-Martin [54], the Bare-eyed Pigeon may flourish on the island as tolerance for urbanization is one of the main drivers of invasion success in birds [55], particularly in Columbidae [48,51]. Although agriculture is limited on Saint-Martin [21], the species is known to cause severe damage to crops in its native range [25], such that an increase in numbers may cause some human–wildlife conflict. In addition, the expansion of the Bare-eyed Pigeon might not be limited to Saint-Martin if some individuals manage to reach neighboring islands in the range of their flying ability, such as Anguilla or Saint-Barthélemy, which are distances of about 19 and 30 km, respectively, from Saint-Martin. In addition, hurricanes, which are relatively frequent in the Caribbean, can facilitate their movement over long distances, as already observed for other Caribbean *Patagioenas* spp. [56]. As the species is well adapted to arid and semi-arid environments [57], its regional expansion might also be further favored by climate change [5], since climate models predict a significant drying in the insular Caribbean during the 21st century [58].

## 5. Conclusions

Successful management of invasive species critically depends on early detection and engagement of stakeholders [59,60]. Failure to respond quickly can lead to a situation beyond human control, as recently happened on the Caribbean island of Barbados, where the introduced Eurasian Collared-Dove, *Streptopelia decaocto*, has rapidly spread across the island since the beginning of the 21st century to become the most dominant dove today, particularly outcompeting the local Zenaida Dove, and the most numerous bird species in some areas of the island. Therefore, we strongly recommend eradicating the exotic Bare-eyed Pigeon on Saint-Martin before it is too late to do so. Regular surveys should be conducted to assess more precisely population numbers and spatial distribution, habitat use, diet, and nesting activity in order to optimize control efforts and evaluate their efficiency. Mist-nests and baited closing net bird traps could be used to capture birds on their feeding grounds, as the technique has previously been used efficiently to capture Columbidae species on other Caribbean islands [61,62]. However, these non-selective techniques are time consuming, such that, based on previous success with eradicating columbid species on islands [63], they should be combined with shooting [64]. In that regard, as the Bare-eyed Pigeon is of potential hunting interest, it could be rapidly added to the official list of game species on Saint-Martin, akin to the Eurasian Collared-Dove. Local hunters should thus be trained to identify Bare-eyed pigeons without ambiguity and be able to differentiate juveniles of the species from those of the White-winged Dove. Control actions should be followed by one or two years of follow-up monitoring to confirm the species’ successful eradication [64]. From that perspective, we recommend close cooperation between administrative authorities on the French and Dutch parts of the island to reinforce control of the local amateur aviculture sector (to limit the risk of repeated introduction) and coordinate eradication and public awareness campaigns. As the risk of rapid spread and fast population growth is very high, control actions should be initiated as early as possible.

## Figures and Tables

**Table 1:** The significance of the model parameters for three logistic regressions. The model (A) analyzes how the probability of detecting more than one individual (Y) varies according to the year, the species, and their interaction. The models (B) and (C) analyze how the probability of detecting more than one Zenaida Dove and Bare-eyed Pigeon varies according to the year, respectively.

Model	Parameter	Odds Ratio [95% CI]	*X* ^2^	*p*
(A) Y ~Year + Species + Year × Species	Year	1.183 [1.016; 1.432]	4.742	0.029
	Species	2.148 [1.388; 3.323]	6.817	0.009
	Year × Species	1.219 [1.047; 1.477]	6.801	0.009
(B) Y ~Year (*Zenaida aurita*)	Year	0.970 [0.935; 1.005]	2.832	0.092
(C) Y ~Year (*Patagioenas corensis*)	Year	1.442 [1.066; 2.112]	5.821	0.016

## Data Availability

Raw data are available on eBird.org.

## Supplementary Materials

The following supporting information can be downloaded via this link: https://www.mdpi.com/article/10.3390/biology13080585/s1. Table S1: List of identification codes corresponding to eBird count events performed by authors.

## Appendix A

**Table A1:** Factor loadings of the four types of land cover on components 1 (PC 1) and 2 (PC 2). See Figure 4 for the PCA plots.

Habitat	PC1	PC2
Tree cover	0.657	−0.277
Open habitat	0.427	0.895
Wetland habitat	−0.994	−0.103
Anthropomorphized habitat	0.595	−0.508
Eigenvalues	1.956	1.146
Cumulative var (%)	48.894	28.656
