# Supplementary material for: Using Citizen Science and Field Surveys to Document the Introduction, Establishment, and Rapid Spread of the Bare-Eyed Pigeon, Patagioenas corensis, on the Island of Saint-Martin, West Indies
Source: Biology (Basel). 2024 Aug 1;13(8):585. doi: 10.3390/biology13080585 (PMC11351522; doi:10.3390/biology13080585)
Supplement: Supplementary file 1 [file biology-13-00585-s001.zip › biology-3127332-supplementary.pdf]

Supplementary materials

Using Citizen Science and Field Surveys to Document the Introduction, Establishment, and Rapid Spread of the Bare-Eyed Pigeon, *Patagioenas corensis*, on the Island of Saint-Martin, West Indies

Christopher Cambrone [1,*], Anthony Levesque [2] and Frank Cézilly [1]

[1] Caribaea Initiative, Le Raizet, 97139 Les Abymes, Guadeloupe, France
[2] Association Amazona, Pointe d'Or, 97139 Les Abymes, Guadeloupe, France
* Correspondence: christopher.cambrone@caribaea.org

Table S1. List of identification codes corresponding to ebird counts carried out by the authors. The date format is dd/mm/yyyy, and the time is in 24h format (hh:mm:ss).

Locality ID	Latitude	Longitude	Date	Time	Sampling event ID
L25723664	18.0459499	-63.0216681	05/07/2023	18:15:00	S143838414
L25723643	18.0499064	-63.0338323	06/07/2023	05:41:00	S143838413
L25723714	18.0556528	-63.0402689	06/07/2023	05:51:00	S143838412
L25723841	18.0732841	-63.0319677	06/07/2023	06:02:00	S143838453
L25724009	18.0763895	-63.0238526	06/07/2023	06:24:00	S143838452
L25724009	18.0763895	-63.0238526	06/07/2023	06:35:00	S143838451
L25724185	18.0787281	-63.0164993	06/07/2023	06:44:00	S143838450
L25724556	18.0835714	-63.0223365	06/07/2023	06:56:00	S143838449
L25724637	18.0893879	-63.0288152	06/07/2023	07:22:00	S143838448
L25724902	18.0911055	-63.0263546	06/07/2023	07:43:00	S143838446
L25725148	18.0966390	-63.0264066	06/07/2023	07:59:00	S143838445
L25734435	18.1147505	-63.0208616	06/07/2023	08:23:00	S143838438
L25725991	18.1051245	-63.0285992	06/07/2023	08:48:00	S143838444
L25726145	18.1081815	-63.0407918	06/07/2023	09:06:00	S143838443
L25726792	18.0910259	-63.0775585	06/07/2023	09:33:00	S143838442
L25727028	18.0891579	-63.0737902	06/07/2023	09:51:00	S143838441
L25727658	18.0889095	-63.0708782	06/07/2023	10:19:00	S143838440
L25735051	18.0930936	-63.0745073	06/07/2023	10:35:00	S143838433
L25734424	18.0476997	-63.0205607	06/07/2023	16:03:00	S143838439
L25734637	18.0543694	-63.0190496	06/07/2023	16:16:00	S143838436
L25734792	18.0572312	-63.0157972	06/07/2023	16:27:00	S143838435
L25735022	18.0623477	-63.0152956	06/07/2023	16:36:00	S143838434
L25735314	18.0662208	-63.0194248	06/07/2023	16:54:00	S143838432
L25735574	18.0698857	-63.0282966	06/07/2023	17:03:00	S143838431
L25735998	18.0468467	-63.0419101	06/07/2023	17:32:00	S143838429
L4265824	18.0265675	-63.0416965	06/07/2023	17:54:00	S143838426
L25723664	18.0459499	-63.0216681	07/07/2023	05:57:00	S143838427
L25743828	18.0458132	-63.0344667	07/07/2023	06:12:00	S143838428
L25744383	18.0927459	-63.0755544	07/07/2023	06:48:00	S143838425

L25726792	18.0910259	-63.0775585	07/07/2023	07:15:00	S143838424

Locality ID	Latitude	Longitude	Date	Time	Sampling event ID
L25744755	18.0847001	-63.0762042	07/07/2023	07:30:00	S143838423
L3679728	18.0727875	-63.1174475	07/07/2023	08:13:00	S143838421
L25745721	18.0645141	-63.1324885	07/07/2023	08:39:00	S143838420
L25745926	18.0709369	-63.1369625	07/07/2023	08:51:00	S143838419
L25746152	18.0650880	-63.1493651	07/07/2023	09:04:00	S143838418
L25746425	18.0613245	-63.1461555	07/07/2023	09:14:00	S143838417
L25752324	18.0636122	-63.1319991	07/07/2023	09:44:00	S143838416
L25754105	18.0688766	-63.0358688	07/07/2023	15:24:00	S143838410
L25723841	18.0732841	-63.0319677	07/07/2023	15:27:00	S143838411
L25754598	18.0961207	-63.0391003	07/07/2023	15:47:00	S144014748
L25754765	18.1040351	-63.0523097	07/07/2023	15:55:00	S143838408
L25755013	18.1023772	-63.0483496	07/07/2023	16:08:00	S143838407
